# In Silico Virtual Screening of Marine Aldehyde Derivatives from Seaweeds against SARS-CoV-2

**DOI:** 10.3390/md20060399

**Published:** 2022-06-16

**Authors:** Nalae Kang, Seong-Yeong Heo, Seon-Heui Cha, Ginnae Ahn, Soo-Jin Heo

**Affiliations:** 1 Jeju Marine Research Center, Korea Institute of Ocean Science and Technology (KIOST), Jeju 63349, Korea; nalae1207@kiost.ac.kr (N.K.); syheo@kiost.ac.kr (S.-Y.H.); 2 Department of Marine Bio and Medical Sciences, Hanseo University, Seosan 31962, Korea; sunnycha@hanseo.ac.kr; 3 Department of Integrated of Bioindustry, Hanseo University, Seosan 31962, Korea; 4 Department of Food Technology and Nutrition, Chonnam National University, Yeosu 59626, Korea; gnahn@jnu.ac.kr; 5 Department of Biology, University of Science and Technology (UST), Daejeon 34113, Korea

**Keywords:** SARS-CoV-2, Coronavirus disease 2019, seaweed, aldehyde derivatives, in silico, virtual screening

## Abstract

Coronavirus disease 2019, caused by the outbreak of severe acute respiratory syndrome coronavirus 2 (SARS-CoV-2), is an ongoing global pandemic that poses an unprecedented threat to the global economy and human health. Several potent inhibitors targeting SARS-CoV-2 have been published; however, most of them have failed in clinical trials. This study aimed to assess the therapeutic compounds among aldehyde derivatives from seaweeds as potential SARS-CoV-2 inhibitors using a computer simulation protocol. The absorption, distribution, metabolism, excretion, and toxicity (ADME/Tox) properties of the compounds were analyzed using a machine learning algorithm, and the docking simulation of these compounds to the 3C-like protease (Protein Data Bank (PDB) ID: 6LU7) was analyzed using a molecular docking protocol based on the CHARMm algorithm. These compounds exhibited good drug-like properties following the Lipinski and Veber rules. Among the marine aldehyde derivatives, 4-hydroxybenzaldehyde, 3-hydroxybenzaldehyde, 3,4-dihydroxybenzaldehyde, and 5-bromoprotocatechualdehyde were predicted to have good absorption and solubility levels and non-hepatotoxicity in the ADME/Tox prediction. 3-hydroxybenzaldehyde and 3,4-dihydroxybenzaldehyde were predicted to be non-toxic in TOPKAT prediction. In addition, 3,4-dihydroxybenzaldehyde was predicted to exhibit interactions with the 3C-like protease, with binding energies of −71.9725 kcal/mol. The computational analyses indicated that 3,4-dihydroxybenzaldehyde could be regarded as potential a SARS-CoV-2 inhibitor.

## 1. Introduction

Coronavirus disease 2019 (COVID-19) is an infectious disease caused by the severe acute respiratory syndrome coronavirus 2 (SARS-CoV-2) [1,2]. The disease has spread worldwide, leading to the ongoing COVID-19 pandemic, which poses an unprecedented threat to the global economy and human health [1,2]. In addition, the World Health Organization suggested that the outbreak of an unknown pathogen may lead to further public health emergencies following the pandemics caused by Ebola, SARS, and Zika viruses [3,4]. Therefore, efforts to identify potent antiviral materials are crucial. 

SARS-CoV-2 primarily spreads between people through aerosols and exhaled respiratory droplets when talking, breathing, coughing, or sneezing [5]. SARS-CoV-2 is a single-stranded RNA-enveloped virus which enters human cells via the viral spike protein binding to the angiotensin-converting enzyme 2 receptor. After virus entry, the incoming genomic RNA releases, and immediately two large open reading frames (ORFs), ORF1a and ORF1b are translated from the positive strand genomic RNA. The generating polyproteins pp1a and pp1ab are processed into individual nonstructural proteins that form the viral replication and transcription complexes [5,6]. The 3C-like protease is responsible for proteolytic processing of the majority of polyprotein cleavage sites [1,7]. Thus, 3C-like proteases are potential targets for SARS-CoV-2 treatment. 

The global pandemic has led to the rapid growth of artificial intelligence technology in various industries [8,9]. In the pharmaceutical industry, virtual screening technology can minimize the cost and time required for novel drug development [10]. Virtual screening technology, including the screening of absorption, distribution, metabolism, excretion, and toxicity (ADME/Tox) properties and molecular docking, is crucial for identifying promising compounds for in vitro and in vivo testing [11]. Safe drugs exhibit a fine-tuned combination of pharmacokinetic and pharmacodynamic variables such as ADME/Tox properties [12]. New drugs which interact with the target proteins to produce therapeutic effects, typically fail in clinical trials because of unfavorable ADME/Tox properties [12,13]. Drugs with several side effects cannot be used commercially and cause enormous financial losses. Therefore, efforts are being made to predict ADME/Tox properties from drug structures in silico at an early development stage [14]. ADME/Tox modeling can aid in the success of drugs in clinical trials [13]. In addition, molecular-docking-based virtual screening identifies compounds with the highest binding affinities and correct binding modes [7,15]. 

Marine organisms have the ability to produce novel bioactive natural products with wide structural diversity, various important human health benefits, and pharmacological potential [16,17]. Seaweeds are photosynthetic organisms that are rich in bioactive materials such as polysaccharides, proteins, peptides, amino acids, and secondary metabolites, including polyphenolic compounds and natural pigments [18]. These bioactive materials have demonstrated various biological activities, including medicinal and health benefits, which has led to an increased demand for these compounds in food, nutraceutical, and cosmeceutical products [19]. Aldehydes are volatile biochemicals which are produced by seaweeds, have low molecular weights, are mostly lipophilic, and are considered the most important parameters of food flavor and quality [20,21]. Aromatic aldehydes have also been reported to possess a wide range of potential bioactive properties including anti-cancer, antibacterial, antioxidant, anti-inflammation, and immunomodulatory effects [22,23,24,25,26,27]. 

The present study aimed to assess the therapeutic compounds among the aldehyde derivatives from seaweeds as potential SARS-CoV-2 inhibitors using a computer simulation protocol. The ADME/Tox properties of the marine aldehyde derivatives were analyzed using a machine learning algorithm and subsequently prioritized based on these results. Next, the docking simulation of the marine aldehyde derivatives to the 3C-like protease (Protein Data Bank (PDB) ID: 6LU7) was analyzed using a molecular docking protocol based on the CHARMm algorithm, and the compounds exhibiting good interactions were selected. 

## 2. Results and Discussion

### 2.1. Drug-Likeness Analysis of the Marine Aldehyde Derivatives

Eleven marine aldehyde derivatives were tested using Lipinski and Veber rules. These aldehyde derivatives exhibited good drug-like properties, based on the number of hydrogen bond acceptors, hydrogen bond donors, molecular weights, ALogP, rotatable bonds, and polar surface areas (Table 1 and Table 2). Compounds with low molecular weights have shown drug-like properties. Therefore, these marine aldehyde derivatives can be considered for the development of new drugs. 

### 2.2. ADME/Tox Analysis of the Marine Aldehyde Derivatives

The 11 aldehyde derivatives were tested in the ADME/Tox Discovery Studio 2021 protocol. In addition, the 2D polar surface area (PSA_2D) for each marine aldehyde derivative was plotted against the corresponding calculated atom-type partition coefficient (ALogP98). 

**Figure 1:**
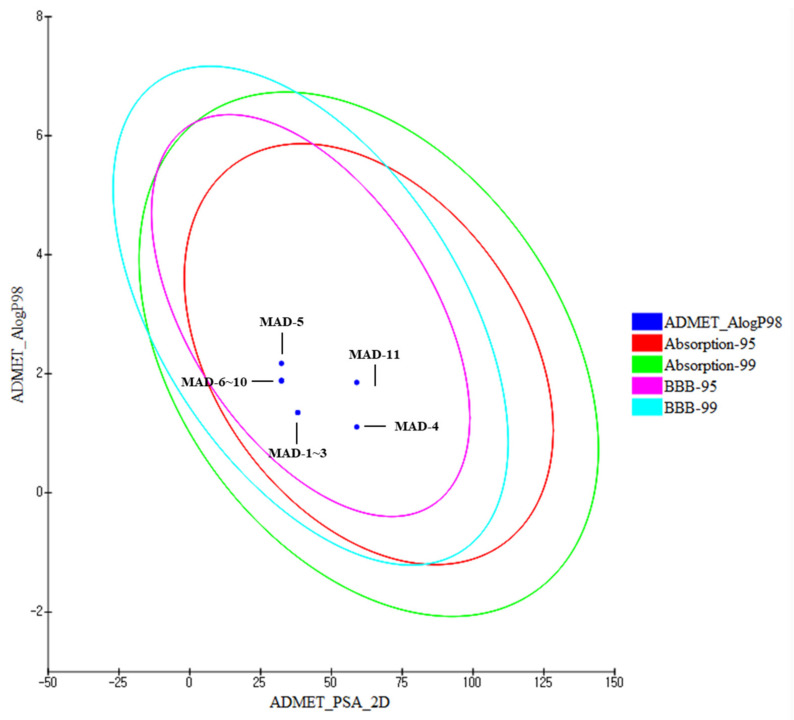
ADMET_AlogP98 and ADMET_PSA_2D attribute graph for the marine aldehyde derivatives. Plot of PSA_2D versus ALogP98 for the marine aldehyde derivatives showing the 95 and 99% confidence limit ellipses corresponding to HIA and BBB models.

The ADME/Tox properties of marine aldehyde derivatives are listed in Table 3. The marine aldehyde derivatives displayed good absorption, solubility, blood–brain barrier (BBB) permeability, and cytochrome P450 2D6 (CYP2D6) prediction levels. All the aldehyde derivatives in this study were located in the human intestinal absorption (HIA) 99% confidence ellipse, and the absorption grade indicated that all the compounds exhibited good absorption (Table 3, Figure 1). In addition, the solubility results indicated that all these compounds exhibited optimal or good solubility (Table 3, Table S1 and Table S2). Drug failure is generally caused by insufficient absorption and distribution of the drug due to low solubility [15]. Thus, marine aldehyde derivatives could be selected as new drug candidates because of their valuable absorption and solubility levels. BBB grade predictions indicated that all the aldehyde derivatives had medium or low BBB permeability, except for MAD-5. These compounds were located in the 99% confidence limit ellipses corresponding to the BBB (Table 3, Table S3 and Table S4, Figure 1). Thus, these marine aldehyde derivatives cannot be used to target nervous systems. CYP2D6 is one of cytochrome P450 enzymes catalyzing the metabolism of the most clinically important drugs. CYP2D6 inhibitors induce a drug–drug interaction, a reaction between two or more other drugs [28]. The CYP2D6 inhibition predictions revealed that none of the marine aldehyde derivatives inhibited the enzyme; therefore, these compounds cannot cause serious drug–drug interaction toxicity. The hepatotoxicity predictions revealed that among the 11 aldehyde derivatives, MAD-1, MAD-2, MAD-4, and MAD-11 exhibited safe hepatotoxicity grades. Drug activity is related to the free drug concentration, which is the drug concentration available for physiological interaction; therefore, it is necessary to determine whether the drug candidates may bind to plasma proteins [29]. The four selected marine aldehyde derivatives exhibited low plasma protein binding activity, with binding rates of <90% (Table 3). Collectively, the results indicate that these four aldehyde derivatives have the potential to be used as new drugs, whereas the remaining aldehyde derivatives require structural optimization before being used in new drugs.

### 2.3. TOPKAT Analysis of Marine Aldehyde Derivatives

Toxicological properties of 11 marine aldehyde derivatives were predicted in silico using the TOPKAT wizard and the 2D molecular structures of the compounds. TOPKAT uses a quantitative structure–toxicity relationship model to assess specific toxicological endpoints [30]. Aspirin and curcumin were selected as positive controls in the TOPKAT analysis. Aspirin, known as acetylsalicylic acid, is a drug used worldwide. Curcumin, a phenolic pigment, is an U.S. Food and Drug Administration-approved drug, and its effect on 3C-like protease of SARS-CoV-2 was evaluated through molecular docking analysis in the previous study [7]. The TOPKAT analysis results for 11 marine aldehyde derivatives are presented in Table 4. Among the aldehyde derivatives, MAD-1, MAD-2, MAD-3, MAD-4, and MAD-11 were predicted to be non-mutagenic according to the Ames test. These five aldehyde derivatives displayed rat oral median lethal doses (LD_50_) ranging between 1.0018 and 2.67949 g/kg body weight (BW). The lowest LD_50_ was exhibited by MAD-3 and the highest LD_50_ by MAD-4. MAD-4 and MAD-11 exhibited higher safe doses compared to those for aspirin, a commercial drug (1.57076 g/kg BW). The rat inhalational median lethal concentration (LC_50_) was predicted for the five aldehyde alternatives. Similarly, MAD-3 displayed the lowest LC_50_ (1655.42 mg/m^3^/h). In addition, MAD-1, MAD-2, MAD-4, and MAD-11 LC_50_ values were predicted to be 1744.04, 2660.83, 1794.97, and 1975.31 mg/m^3^/h, respectively. Curcumin displayed a LC_50_ value of 1200.8 mg/m^3^/h, implying that the four aldehyde derivatives are safer compared to curcumin. MAD-3 was excluded from further analyses because of its low LD_50_ and LC_50_. Next, skin irritancy evaluation indicated that MAD-2, MAD-4, and MAD-11 were absent in the skin, whereas MAD-1 was present. In addition, the three aldehyde derivatives which were absent from the skin were predicted to be non-carcinogenic in both female and male rats according to the US National Toxicology Program (NTP) model, except for MAD-11, which revealed potential carcinogenicity in the male rat NTP. Therefore, the TOPKAT prediction results indicated that MAD-2 and MAD-4 are expected to display the best pharmacokinetic and pharmacodynamic behaviors with no mutagenic, carcinogenic, or irritant effects; thus, MAD-2 and MAD-4 are potential SARS-CoV-2 inhibitors.

### 2.4. Molecular Docking Analysis of Marine Aldehyde Derivatives on 3C-like Protease

Several molecular docking studies targeting specific proteins, including enzymes and receptors, have recently been published [7,18,31,32,33]. Among the docking tools, CDOCKER, a CHARMm-based docking algorithm, found favorable docking poses between small molecules and target proteins based on their structural characteristics such as unshared electron pairs, double bonds, hydrophobicity, and charge [34].

**Figure 2:**
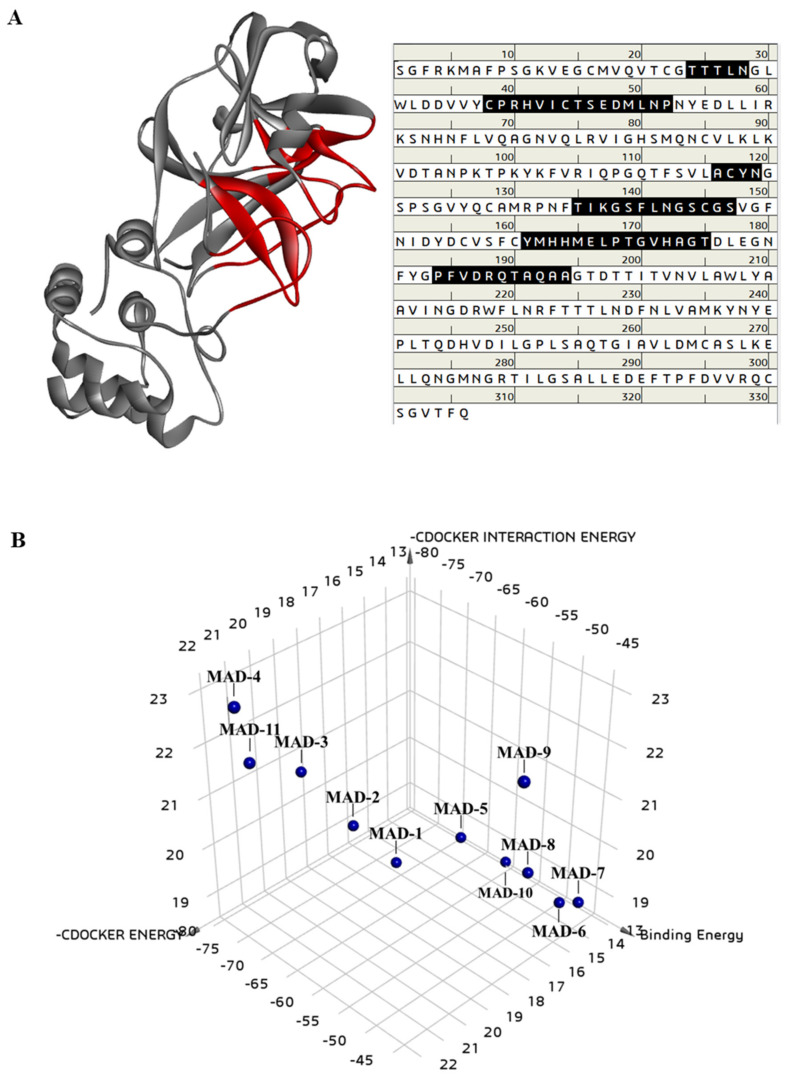
Molecular docking analysis for the marine aldehyde derivatives to the 3C-like protease of SARS-CoV-2. Preparation of 3C-like protease 3D structure (**A**). Binding site (red color) and amino acid sequence (black color). The 2D chart of the docking poses of the marine aldehyde derivatives to the 3C-like protease expressed as –CDOCKER energy, -CDOCKER interaction, and binding energies (**B**).

The inhibitory effects of marine aldehyde derivatives on the 3C-like protease of SARS-CoV-2 were predicted by simulating the biological network dynamics of the marine aldehyde derivatives and 3C-like protease in a computational space. The binding energies of the compounds to the 3-C like protease were compared with that of curcumin as a positive control, following a previous study [7,35]. The crystal structure of the 3C-like protease was obtained from the Protein Data Bank (PDB ID 6LU7, [1]), and the structure was confirmed and revised through structural optimization. The binding site was analyzed using the binding sphere of the already docked inhibitor N3 to the 3C-like protease. The amino acids in the binding site are composed as follows: THR24, THR25, THR26, LEU27, ASN28, CYS38, PRO39, ARG40, HIS41, VAL42, ILE43, CYS44, THR45, SER46, GLU47, ASP48, MET49, LEU50, ASN51, PRO52, ALA116, CYS117, TYR118, ASN119, THR135, ILE136, LYS137, GLY138, SER139, PHE140, LEU141, ASN142, GLY143, SER144, CYS145, GLY146, SER147, TYR161, MET162, HIS163, HIS164, MET165, GLU166, LEU167, PRO168, THR169, GLY170, VAL171, HIS172, ALA173, GLY174, THR175, PRO184, PHE185, VAL186, ASP187, ARG188, GLN189, THR190, ALA191, GLN192, ALA193, and ALA194 (Figure 2A). The structures of marine aldehyde derivatives and curcumin were downloaded from PubChem and the 3D structures were optimized.

The binding pattern of the marine aldehyde derivatives to the 3C-like protease were analyzed, and these docking poses were expressed as a 3D chart using -CDOCKER interaction energy (kcal/mol), -CDOCKER energy (kcal/mol), and binding energy (kcal/mol) (Figure 2B and Table 5). Among the marine aldehyde derivatives, MAD-4 bound most stably to the 3C-like protease, with the highest -CDOCKER energy (22.4808 kcal/mol) and -CDOCKER interaction energy (23.2915 kcal/mol), and the lowest binding energy (−71.9725 kcal/mol). In succession, MAD-11, MAD-2, and MAD-1, which were predicted to exhibit favorable ADME/Tox or TOPKAT levels, displayed relatively low binding energies compared to that for other marine aldehyde derivatives (Figure 2B and Table 5). In particular, these four marine aldehyde derivatives were docked to the active site of the 3C-like protease and displayed lower binding energies than that of curcumin, which was used as a positive control (Table 5). This implies that these aldehyde derivatives could bind more stably to the 3C-like protease compared to that seen with curcumin.

**Figure 3:**
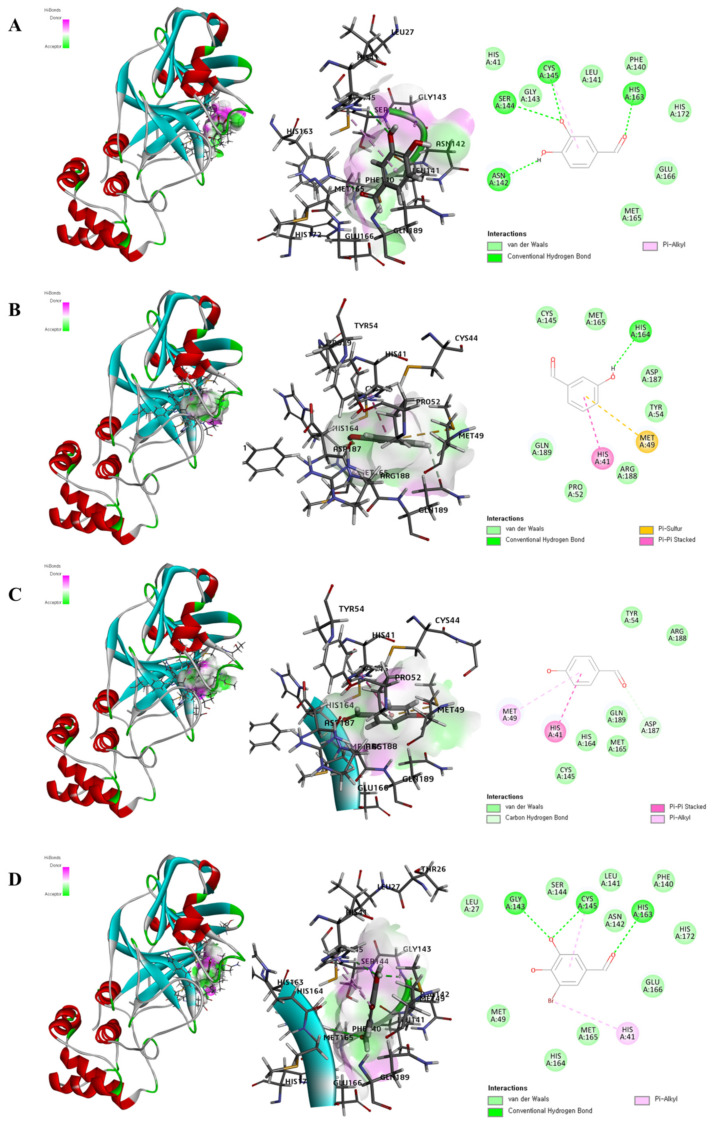
The docking poses of the marine aldehyde derivative-3C-like protease of SARS-CoV-2. The 3D and 2D diagram of the complexes to 3C-like protease with MAD-4 (**A**), MAD-2 (**B**), MAD-1 (**C**), and MAD-11 (**D**). The 3C-like protease was expressed as a ribbon model tagging the amino acid. The marine aldehyde derivatives are shown as a gray and red stick model, and the binding surface is expressed in terms of hydrogen bonds. The 2D diagram of the marine aldehyde derivative–3C-like protease complexes were combined as hydrogen bond and/or pi bond.

Each oxygen molecule in MAD-4 formed four hydrogen bonds with ASN142, SER144, CYS145, and HIS163. The phenol ring of MAD-4 formed a pi bond with CYS145 (Figure 3A). MAD-2 bound to the 3C-like protease exhibiting –CDOCKER energy of 17.2844 kcal/mol, –CDOCKER interaction energy of 19.576 kcal/mol, and the lowest binding energy of −74.1383 kcal/mol. The hydrogen in MAD-2 formed hydrogen bonds with HIS164, and the phenol ring of MAD-2 formed a pi-pi stacked bond with HIS41 and a pi-sulfur bond with MET49 (Figure 3B). MAD-1 bound to the 3C-like protease exhibiting −CDOCKER energy of 16.3341 kcal/mol, –CDOCKER interaction energy of 18.8237 kcal/mol, and the lowest binding energy of −69.5871 kcal/mol. The oxygen molecules in MAD-1 formed hydrogen bonds with ASP187, and the phenol ring of MAD-1 formed a pi-pi stacked bond with HIS41 and a pi-alkyl bond with MET49 (Figure 3C). MAD-11 bound stably to the 3C-like protease exhibiting –CDOCKER energy of 21.4484 kcal/mol, –CDOCKER interaction energy of 21.8194 kcal/mol, and the lowest binding energy of −74.9887 kcal/mol. The oxygen molecules in MAD-11 formed hydrogen bonds with GLY143, CYS145, and HIS163, and the phenol ring of MAD-11 formed a pi-alkyl bond with CYS145. The bromine of MAD-11 formed a pi-alkyl bond with HIS41 (Figure 3D). These in silico based results indicated that MAD-4 has the potential to be used as a SARS-CoV-2 inhibitor. Further studies are needed to confirm the inhibitory effects of these marine aldehyde derivatives on SARS-CoV-2 in vitro.

Previous studies have presented structural evidence of the antiviral activities of seaweed polysaccharides [36,37,38,39]. Previous studies have also reported that phlorotannins derived from seaweeds have potential antiviral activities against SARS-CoV, porcine epidemic diarrhea coronavirus, HIV-1, and VHSV [40,41,42,43,44]. However, the antiviral activities of various natural marine products such as aldehyde derivatives, remain unknown.

Marine aldehyde derivatives including indole-4-carboxaldehyde (MAD-7, from *Sargassum thunbergii*), 3,4-dihydroxybenzaldehyde (MAD-4, from *Polysiphonia morrowii*), and 5-bromo-3,4-dihydroxybenzaldehyde (from *P. morrowii*), have been reported to possess various bioactivities, including inhibition of hepatic inflammation [22] and anti-allergy [23,26], and hair growth effects [24]. An aromatic aldehyde, 3-chloro-4,5-dihydroxybenzaldehyde, has been shown to inhibit adipogenesis in adipocytes [25]. Additionally, 5-bromo-2-hydroxy-4-methyl-benzaldehyde has been shown to exhibit anti-inflammatory activities via the inactivation of the ERK, p38, and NF-κB pathways [27]. These studies suggest that marine aldehyde derivatives have pharmaceutical potential. According to the drug-like properties predictions of the 11 marine aldehyde derivatives, these compounds also are likely to have good drug-like properties for commercial use. In addition, the marine aldehyde derivatives tested in this study, including MAD-4 exhibit viral inhibition potential through diverse cell signaling pathways. Future research may investigate the various antiviral activities of marine aldehyde derivatives via in vitro studies.

## 3. Materials and Methods

### 3.1. 3D Structure of Proteins and the Marine Aldehyde Derivatives

For molecular docking studies, the crystal structure of the 3C-like protease (PDB ID: 6LU7) was obtained from the Protein Data Bank. The crystal structure had a resolution of 2.16 Å; therefore, it was used in this computational study. The “prepare protein” protocols of the Discovery Studio 2021 tool were applied for protein structure preparation. The binding site of the 3C-like protease was defined from the current docking site of the N3 ligand, following a previous paper [3]. The 3D structures of 11 marine aldehyde derivatives were obtained from PubChem. The compound names and PubChem compound ID (CID) numbers are as follows: 4-hydroxybenzaldehyde (CID: 126), 3-hydroxybenzaldehyde (CID: 101), salicylaldehyde (CID: 6998), 3,4-dihydroxybenzaldehyde (CID: 8768), indole-2-carboxaldehyde (CID: 96389), indole-3-carboxaldehyde (CID: 10256), indole-4-carboxaldehyde (CID: 333703), indole-5-carboxaldehyde (589040), indole-6-carboxaldehyde (CID: 2773435), indole-7-carboxaldehyde (CID: 2734629), and 5-bromoprotocatechualdehyde (CID: 85405) (Table 1). The geometry optimization of the marine aldehyde derivatives was performed using the BIOVIA Discovery Studio 2021 protocol of energy minimization and numbered the compounds in order from MAD-1 to MAD-11 (Table 2).

### 3.2. Drug-like Properties and ADME/Tox Predictions of the Marine Aldehyde Derivatives

Marine aldehyde derivatives were subjected to drug-like properties and ADME/Tox profiling using Discovery Studio 2021 (Biovia, San Diego, CA, USA). The drug-like properties of the marine aldehyde derivatives were predicted as follows: Lipinski rules (number of hydrogen bond donors < 5, number of hydrogen bond acceptors < 10, molecular weight < 500 Da, ALogP < 5, and no more than one violation of the above criteria) and Veber rules (rotatable bonds < 10, Polar Surface Area < 140, hydrogen bond donors and acceptors < 12) [45,46]. The ADME/Tox properties of the marine aldehyde derivatives were predicted using the Discovery Studio 2021 Descriptor module: six mathematical models, including HIA level, aqueous solubility level, BBB level, CYP2D6 prediction, hepatotoxicity prediction, and PPB prediction. The ADME/Tox protocol of Discovery Studio 2021 defines a perfect drug candidate as exhibiting good absorption, optimal/good/low solubility, medium/low BBB permeability, non-inhibitor/inhibitor CYP2D6, non-toxic hepatotoxicity, and <90% PPB [47]. The reliability of the predictions was analyzed via the HIA 99% confidence ellipse and BBB 99% confidence ellipse.

### 3.3. TOPKAT Predictions of Marine Aldehyde Derivatives

The toxicity of the marine aldehyde derivatives was predicted using the TOPKAT module of Discovery Studio 2021 (Biovia, San Diego, CA, USA). The chosen parameters in the TOPKAT prediction were the Ames test for mutagenicity, rat oral LD_50_, rat inhalational LC_50_, skin irritation, and female and male rat NTP. Results from these tests were used to select compounds with considerable pharmacokinetic behavior and low toxicity.

### 3.4. Molecular Docking Analysis of the Marine Aldehyde Derivatives on the 3C-like Protease

CDOCKER docking was performed to assess the binding poses of the marine aldehyde derivatives within the active site of the 3C-like protease. Molecular docking analysis was performed using the CDOCKER and Calculate Binding Energies tools in Discovery Studio 2021 (Biovia, San Diego, CA, USA). In particular, a docking mechanism based on CHARMm [34] was used to execute the docking protocol for CDOCKER. Docking of marine aldehyde derivatives to the 3C-like protease was performed as follows: (1) a 2D structure was converted into a 3D structure; (2) proteins were prepared, and the binding site was defined; and (3) docking of compounds was performed using the CDOCKER tool [32]. The binding pocket of the 3C-like protease was assigned as the area from the center of the active site within a radius of 14.8 Å. The binding site and ligand were allowed to move freely during docking. The water molecules are removed from the protein in the flexible docking process because the fixed water molecules might alter the generation of the ligand–receptor complex. After the removal of water molecules, hydrogen atoms were attached to the protein. The ligand-binding affinity was assessed for all complexes by applying the CHARMm force field to the interaction energy. Based on CDOCKER’s interaction energy, distinct conformational poses for each molecule were produced and examined. The binding energies of the produced small-molecule–protein complexes were calculated using the Calculate Binding Energies tool. Three types of energy values (–CDOCKER interaction energy, –CDOCKER energy, and binding energy) were generated for the produced complexes; these were used to select candidate compounds. The docking positions of the selected marine aldehyde derivatives to the 3C-like protease were expressed as 2D diagrams and 3D crystal structures.

## 4. Conclusions

Marine aldehyde derivatives have good drug-like properties following Lipinski and Veber rules. MAD-4 and MAD-2 were selected for further study following in silico bioavailability and toxicity results. These compounds were predicted to interact more strongly with the 3C-like protease than curcumin, with binding energies of −74.1383 and −71.9725 kcal/mol, respectively. In addition, MAD-4 displayed higher −CDOCKER energy and −CDOCKER interaction energy. This study suggests that the drug discovery approach used provides insight into therapeutics that might be helpful in treating COVID-19. These in silico based results indicate that MAD-4 has the potential to be used as a SARS-CoV-2 inhibitor. Future research utilizing in vitro studies can be performed to confirm the ADME/Tox levels and antiviral-activity predictions of the marine aldehyde derivatives.

## Figures and Tables

**Table 1:** The marine aldehyde derivatives list.

**3D structure**	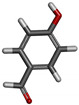	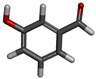
**Chemical name**	4-hydroxybenzaldehyde	3-hydroxybenzaldehyde
**MAD no.**	MAD-1	MAD-2
**3D structure**	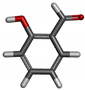	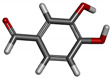
**Chemical name**	Salicylaldehyde	3,4-dihydroxybenzaldehyde
**MAD no.**	MAD-3	MAD-4
**3D structure**	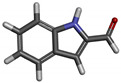	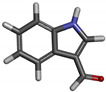
**Chemical name**	indole-2-carboxaldehyde	indole-3-carboxaldehyde
**MAD no.**	MAD-5	MAD-6
**3D structure**	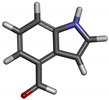	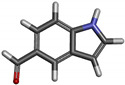
**Chemical name**	indole-4-carboxaldehyde	indole-5-carboxaldehyde
**MAD no.**	MAD-7	MAD-8
**3D structure**	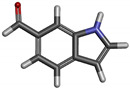	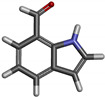
**Chemical name**	indole-6-carboxaldehyde	indole-7-carboxaldehyde
**MAD no.**	MAD-9	MAD-10
**3D structure**	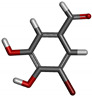	
**Chemical name**	5-bromoprotocatechualdehyde	
**MAD no.**	MAD-11	

**Table 2:** In silico analysis of drug-like properties of the marine aldehyde derivatives.

Marine Aldehyde Derivatives	HBD	HBA	MW (Da)	ALogP	RB	PSA
**MAD-1**	1	2	122.121	1.347	1	37.29
**MAD-2**	1	2	122.121	1.347	1	37.29
**MAD-3**	1	2	122.121	1.347	1	37.29
**MAD-4**	2	3	138.121	1.105	1	57.53
**MAD-5**	1	2	145.158	2.174	1	32.86
**MAD-6**	1	2	145.158	1.882	1	32.86
**MAD-7**	1	2	145.158	1.882	1	32.86
**MAD-8**	1	2	145.158	1.882	1	32.86
**MAD-9**	1	2	145.158	1.882	1	32.86
**MAD-10**	1	2	145.158	1.882	1	32.86
**MAD-11**	2	3	217.017	1.853	1	57.53

HBD, hydrogen bond donors; HBA, hydrogen bond acceptors; RB, rotatable bonds; PSA, polar surface area.

**Table 3:** In silico based ADME/Tox analysis of the marine aldehyde derivatives.

Marine Aldehyde Derivatives	AL	SL	BL	CP	HP	PP
**MAD-1**	Good	Optimal	Medium	Non-inhibitor	Non-toxic	Binding is <90%
**MAD-2**	Good	Optimal	Medium	Non-inhibitor	Non-toxic	Binding is <90%
**MAD-3**	Good	Optimal	Medium	Non-inhibitor	Toxic	Binding is >90%
**MAD-4**	Good	Optimal	Low	Non-inhibitor	Non-toxic	Binding is <90%
**MAD-5**	Good	Good	High	Non-inhibitor	Toxic	Binding is >90%
**MAD-6**	Good	Good	Medium	Non-inhibitor	Toxic	Binding is <90%
**MAD-7**	Good	Good	Medium	Non-inhibitor	Toxic	Binding is <90%
**MAD-8**	Good	Good	Medium	Non-inhibitor	Toxic	Binding is <90%
**MAD-9**	Good	Good	Medium	Non-inhibitor	Toxic	Binding is <90%
**MAD-10**	Good	Good	Medium	Non-inhibitor	Toxic	Binding is >90%
**MAD-11**	Good	Optimal	Medium	Non-inhibitor	Non-toxic	Binding is <90%

AL, absorption level; SL, solubility level; BL, BBB level; CP, CYP2D6 prediction; HP, hepatotoxic prediction; PP, PPB prediction.

**Table 4:** TOPKAT analysis of the marine aldehyde derivatives.

Marine Aldehyde Derivatives	Ames Mutagenicity	Rat Oral LD_50_ (g/kg BW)	Rat Inhalational LC_50_ (mg/m^3^/h)
MAD-1	Non-Mutagen	1.13365	1744.04
MAD-2	Non-Mutagen	1.31137	2660.83
MAD-3	Non-Mutagen	1.0018	1655.42
MAD-4	Non-Mutagen	2.67949	1794.97
MAD-5	Mutagen	0.68308	4020.58
MAD-6	Mutagen	0.393331	2431.62
MAD-7	Mutagen	0.213938	2431.62
MAD-8	Mutagen	0.535304	4117.71
MAD-9	Mutagen	0.535304	4117.71
MAD-10	Mutagen	0.551856	2431.62
MAD-11	Non-Mutagen	1.96303	1975.31
Asprin	Non-Mutagen	1.57076	2704.1
Curcumin	Non-Mutagen	2.81353	1200.8
			
**Marine aldehyde** **derivatives**	**Skin** **Irritancy**	**Female Rat** **NTP**	**Male Rat NTP**
MAD-1	Mild	Non-Carcinogen	Non-Carcinogen
MAD-2	None	Non-Carcinogen	Non-Carcinogen
MAD-3	None	Non-Carcinogen	Non-Carcinogen
MAD-4	None	Non-Carcinogen	Non-Carcinogen
MAD-5	Mild	Carcinogen	Carcinogen
MAD-6	Mild	Non-Carcinogen	Carcinogen
MAD-7	Mild	Non-Carcinogen	Non-Carcinogen
MAD-8	Mild	Non-Carcinogen	Non-Carcinogen
MAD-9	Mild	Non-Carcinogen	Non-Carcinogen
MAD-10	Mild	Non-Carcinogen	Non-Carcinogen
MAD-11	None	Non-Carcinogen	Carcinogen
Asprin	None	Non-Carcinogen	Non-Carcinogen
Curcumin	Mild	Carcinogen	Non-Carcinogen

**Table 5:** Calculated energies of the marine aldehyde derivatives on the 3C-like protease of SARS-CoV-2.

Marine Aldehyde Derivatives	3C-like Proteinase (6LU7)		
	–CDOCKER Energy (kcal/mol)	–CDOCKER Interaction Energy (kcal/mol)	Binding Energy (kcal/mol)
**MAD-1**	16.3341	18.8237	−69.5871
**MAD-2**	17.2844	19.576	−74.1383
**MAD-3**	18.0852	20.4885	−80.9339
**MAD-4**	22.4808	23.2915	−71.9725
**MAD-5**	14.1641	19.0705	−65.4523
**MAD-6**	13.4791	18.5471	−49.2269
**MAD-7**	13.4622	18.7735	−46.0088
**MAD-8**	14.7365	19.4259	−50.8909
**MAD-9**	17.7453	22.5439	−42.0406
**MAD-10**	12.8445	18.4353	−61.5275
**MAD-11**	21.4484	21.8194	−74.9887
**Curcumin**	35.4411	45.3384	−63.3906

## Data Availability

Not applicable.

## Supplementary Materials

The following supporting information can be downloaded at: https://www.mdpi.com/article/10.3390/md20060399/s1, Table S1: In silico–based aqueous solubility analysis of the marine aldehyde derivatives; Table S2: Key to aqueous solubility levels indicator; Table S3: In silico–based BBB permeability analysis of the marine aldehyde derivatives; Table S4: Key to BBB permeability levels indicator.
