# Supplementary material for: In Silico Virtual Screening of Marine Aldehyde Derivatives from Seaweeds against SARS-CoV-2
Source: Mar Drugs. 2022 Jun 16;20(6):399. doi: 10.3390/md20060399 (PMC9227357; doi:10.3390/md20060399)
Supplement: Supplementary file 1 [file marinedrugs-20-00399-s001.zip › marinedrugs-1761497-supplementary.pdf]

Supplementary materials

Table S1

In silico–based aqueous solubility analysis of the marine aldehyde derivatives.

Marine aldehyde Derivatives	Log Sw	Level	Drug-likeness
**MAD-1**	-1.296	4	Optimal
**MAD-2**	-1.295	4	Optimal
**MAD-3**	-1.292	4	Optimal
**MAD-4**	-0.823	4	Optimal
**MAD-5**	-3.019	3	Good
**MAD-6**	-2.755	3	Good
**MAD-7**	-2.756	3	Good
**MAD-8**	-2.772	3	Good
**MAD-9**	-2.772	3	Good
**MAD-10**	-2.756	3	Good
**MAD-11**	-1.747	4	Optimal

Log Sw, the logarithm of the aqueous solubility

Table S2

Key to aqueous solubility levels indicator.

Level	Value	Drug-likeness
**0**	log(Sw) < -8.0	Extremely low
**1**	-8.0 < log(Sw) < -6.0	No, very low, but possible
**2**	-6.0 < log(Sw) < -4.1	Yes, low
**3**	-4.1 < log(Sw) < -2.0	Yes, good
**4**	-2.0 < log(Sw) < 0.0=””	Yes, optimal
**5**	0.0 < log(Sw)	No, too soluble
**6**	-1000	Warning: molecules with one or more unknown AlogP98 types

Log Sw, the logarithm of the aqueous solubility

Table S3

In silico–based BBB permeability analysis of the marine aldehyde derivatives.

Marine aldehyde Derivatives	Log BB	Level	Value
**MAD-1**	-0.341	2	Medium
**MAD-2**	-0.341	2	Medium
**MAD-3**	-0.341	2	Medium
**MAD-4**	-0.745	3	Low
**MAD-5**	0.006	1	High
**MAD-6**	-0.084	2	Medium
**MAD-7**	-0.084	2	Medium
**MAD-8**	-0.084	2	Medium
**MAD-9**	-0.084	2	Medium
**MAD-10**	-0.084	2	Medium
**MAD-11**	-0.514	2	Medium

Log BB, the logarithmic ratio between the concentration of a drug in the brain and blood

Table S4

Key to BBB permeability levels indicator.

Level	Value	Description
**0**	Very High	log BB ≥ 0.7
**1**	High	0 ≤ log BB < 0.7
**2**	Medium	-0.52 < log BB < 0
**3**	Low	log BB ≤ -0.52
**4**	Undefined	Outside 99% confidence ellipse
**5**	AlogP98	Warning: molecules with one or more unknown AlogP98 types

Log BB, the logarithmic ratio between the concentration of a drug in the brain and blood
